# Histiocytic Sarcoma: A Review and Update

**DOI:** 10.3390/ijms26178554

**Published:** 2025-09-03

**Authors:** Yuki Shinohara, Shizuhide Nakayama, Mikiko Aoki, Jun Nishio

**Affiliations:** 1 Section of Orthopaedic Surgery, Department of Medicine, Fukuoka Dental College, 2-15-1 Tamura, Sawara-ku, Fukuoka 814-0193, Japan; yukis@fdcnet.ac.jp; 2 Department of Orthopaedic Surgery, Faculty of Medicine, Fukuoka University, 7-45-1 Nanakuma, Jonan-ku, Fukuoka 814-0180, Japan; n.shizuhide@gmail.com; 3 Department of Pathology, Faculty of Medicine, Fukuoka University, 7-45-1 Nanakuma, Jonan-ku, Fukuoka 814-0180, Japan; mikikoss@fukuoka-u.ac.jp

**Keywords:** histiocytic sarcoma, malignant histiocytosis, hematopoietic neoplasm, diagnosis, pathogenesis, treatment

## Abstract

Histiocytic sarcoma (HS) is an ultra-rare hematopoietic neoplasm that frequently occurs in extranodal sites of adults. Clinically, HS demonstrates aggressive behavior and can arise de novo or in association with other hematological neoplasms. The median overall survival from the time of diagnosis is approximately six months. Histologically, HS is composed of sheets of large, round to oval cells with abundant eosinophilic cytoplasm and can be confused with a variety of benign and malignant conditions. Immunohistochemistry plays a crucial role in the diagnosis of HS, showing expression of CD163, CD68, lysozyme, and PU.1 and negative staining with follicular dendritic cell markers and myeloid cell markers. Recent studies have demonstrated a high rate of PD-L1 expression, suggesting a potential therapeutic target. Several genomic alterations have been identified in HS, including mutations involving the RAS/MAPK and PI3K/AKT/mTOR signaling pathways, *CDKN2A* mutations/deletions, and *TP53* mutations. There is no standard protocol for the management of HS. Surgical resection with or without radiotherapy is the most common first-line treatment for unifocal/localized disease. The systemic treatment options for multifocal/disseminated disease are very limited. This review provides an overview of the current knowledge on the clinicoradiological features, histopathology, pathogenesis, and management of HS.

## 1. Introduction

Histiocytic sarcoma (HS) is an extremely rare and highly aggressive hematopoietic neoplasm showing morphological and immunophenotypic features of mature histiocytes. According to the latest World Health Organization (WHO) classification of hematolymphoid tumors, HS belongs to the group of histiocyte/macrophage neoplasms [1]. The overall incidence of HS is less than 0.17 per 1,000,000 individuals [2]. The etiology of this disorder is uncertain. Moreover, the natural history of HS is largely unknown. HS can be difficult to diagnose due to its rarity and morphological overlap with diverse mimics. Immunohistochemistry is essential in the diagnosis of HS. Surgery is the treatment of choice for unifocal/localized disease. There is currently no consensus regarding the optimal treatment strategy for multifocal/disseminated disease. Large randomized clinical trials have not been undertaken for HS. This review highlights the clinicoradiological, histological, immunohistochemical, and genomic features of HS. In addition, we will summarize the current management of this ultra-rare neoplasm.

## 2. Clinical Features and Prognosis

HS can occur at any age but has a peak incidence in the sixth to seventh decades of life, with a slight male predominance [2,3,4]. It frequently occurs in extranodal sites, including the soft tissue, skin, respiratory system, gastrointestinal (GI) tract, central nervous system (CNS), and spleen. Lymph node involvement (14%) has also been reported [4]. HS can be localized or disseminated. Extranodal HS typically presents as a painless solitary mass, ranging in size from 1.8 to 12 cm (median 6.8 cm) [5]. Symptoms are related to local compression of surrounding organs or express with constitutional manifestations such as fever, fatigue, night sweats, and weight loss. Lymphadenopathy is often observed. A subset of cases can arise subsequent to or concurrent with hematological neoplasms, including follicular lymphoma (FL), chronic lymphocytic leukemia/small lymphocytic lymphoma (CLL/SLL), and B- or T-lymphoblastic leukemia/lymphoma [1,3,6]. In addition, only a few cases are associated with mediastinal germ cell tumors [7,8]. The risk factors for the development of secondary malignancies are unknown.

Importantly, HS pursues an aggressive clinical course with a limited response to therapy and a high mortality rate [1]. The median overall survival (OS) from the time of diagnosis is approximately six months [2]. Most patients die of progressive disease within two years [3]. Nonetheless, some patients with localized disease may have a favorable long-term outcome [5]. Compared with de novo HS, secondary HS has a significantly worse OS [9]. The potential prognostic factors include age at diagnosis, tumor size, tumor site, elevated lactate dehydrogenase (LDH), patient performance status (PS), patient comorbidities, and stage at presentation [3,4,5,10].

## 3. Imaging Features

The imaging features of HS are non-specific and depend on the site of involvement [11]. Ultrasonography with color Doppler shows a multilobulated, mostly hypoechoic mass with internal vascularity [12,13]. On magnetic resonance imaging (MRI), HS may reveal a well or poorly circumscribed soft tissue mass with low to intermediate signal intensity on T1-weighted images and heterogeneous high signal intensity on T2-weighted images. Contrast-enhanced MRI typically demonstrates diffuse, mostly homogeneous or moderately heterogeneous enhancement of the lesion [13,14].

It is generally recognized that fluorodeoxyglucose (FDG) position emission tomography (PET)/computed tomography (CT) is advantageous for staging and assessing treatment response of HS compared to other imaging modalities [11]. PET/CT shows intense FDG uptake in both nodal and extranodal lesions, with the high maximum standardized uptake value (SUVmax) [14,15,16,17].

## 4. Histopathological and Immunohistochemical Features

Grossly, HS often appears as a fleshy mass with a grayish white cut surface. Hemorrhage and necrosis may be seen [1].

**Figure 1:**
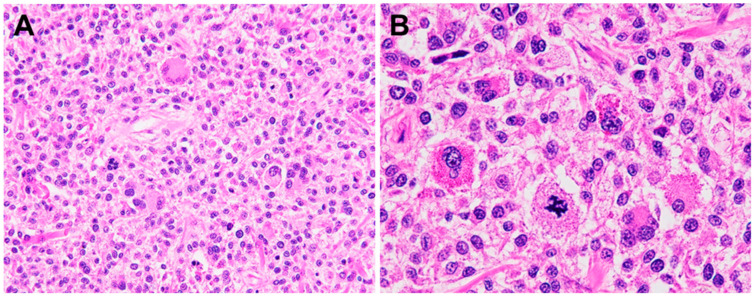
Histopathology of histiocytic sarcoma (HS). (**A**) HS is composed of sheets of large, round to oval cells with abundant eosinophilic cytoplasm. Binucleated or multinucleated cells can be seen. (**B**) Atypical mitosis is easily identified.

HS shows a diffuse infiltration in the soft tissue and a sinusoidal distribution may be seen in lymph node, liver, and spleen [1]. Histologically, HS is composed of sheets of large, round to oval cells with abundant eosinophilic cytoplasm, oval to irregular nuclei with vesicular chromatin, and variably prominent nucleoli (Figure 1). Nuclear atypia is variable. Binucleated or multinucleated cells are commonly observed [5]. Some cases may show significant pleomorphism with focal areas of spindle cells. Hemophagocytosis is occasionally identified within the neoplastic cells. Mitotic activity may be frequent, and necrosis is common. There is usually a prominent inflammatory background, most often consisting of neutrophils and lymphocytes.

**Figure 2:**
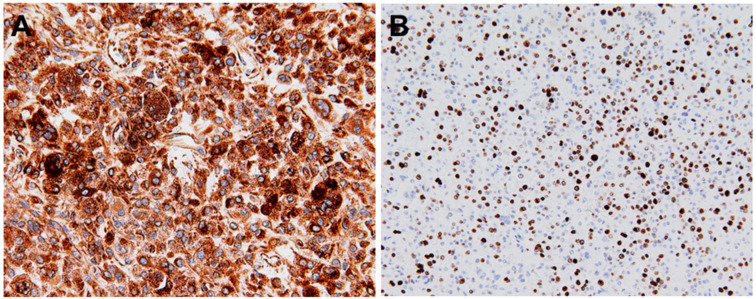
Immunohistochemistry of histiocytic sarcoma (HS). The neoplastic cells are strongly positive for CD68 (**A**) and Ki-67 (**B**).

Immunohistochemically, the neoplastic cells show expression multiple histiocytic markers including CD163, CD68, and lysozyme (Figure 2). In addition, CD4, CD31, CD45, CD45RO, and PU.1 are usually positive [1]. It should be kept in mind that these markers are not specific for HS. Variable expression for S100 is also observed [5]. Most recently, Patwardhan et al. reported that interferon regulatory factor 8 (IRF8) expression was present in 50% (3/6) of HS cases [18]. Immunostainings for specific B-cell and T-cell markers (CD20, PAX5, and CD3), Langerhans cell markers (CD1a and CD207), follicular dendritic cell markers (CD21, CD23, and CD35), myeloid cell markers (CD13, CD33, and myeloperoxidase), melanocytic markers (HMB45, Melanin A, and SOX10), anaplastic lymphoma kinase (ALK), and cytokeratins are typically negative [1]. The Ki-67 index is variable [3].

The histological differential diagnosis of HS is notably broad and includes anaplastic large cell lymphoma (ALCL), Langerhans cell sarcoma (LCS), interdigitating dendritic cell sarcoma (IDCS), follicular dendritic cell sarcoma (FDCS), myeloid sarcoma, melanoma, undifferentiated large cell carcinoma, epithelioid sarcoma, and undifferentiated pleomorphic sarcoma (UPS). In our opinion, ALCL is most frequently confused with HS. ALCL is uniformly positive for CD30 with a variable expression of T-cell markers, whereas histiocytic markers (CD163, CD68, and lysozyme) are negative. LCS may also have overlapping histological features of HS, but it expresses Langerhans cell markers (CD1a and CD207) as well as S100. A subset of IDCSs show focal areas with an epithelioid morphology, mimicking HS. In contrast to HS, IDCS is diffusely and strongly positive for S100 with a weak expression of CD68 and lysozyme. Although FDCS should be considered in the differential diagnosis of HS, it is positive for follicular dendritic cell markers (CD21, CD23, and CD35). Myeloid sarcoma with monocytic differentiation can be confused with HS, but it expresses myeloid cell markers (CD13, CD33, and myeloperoxidase). Additionally, the blasts are much smaller than the HS cells with a higher nuclear/cytoplasmic (N/C) ratio. In our experience, melanoma should be excluded using melanocytic markers such as SOX10 and HMB45, which are typically negative in HS. Metastatic carcinoma, especially undifferentiated carcinoma, occasionally shows abundant eosinophilic cytoplasm, closely resembling HS [5]. However, carcinoma lacks expression of CD163 and is positive for cytokeratins and epithelial membrane antigen (EMA). Epithelioid sarcoma can mimic HS with an epithelioid-to-pleomorphic morphology and prominent tumor necrosis. Unlike HS, however, epithelioid sarcoma is positive for cytokeratins and EMA, along with loss of SWI/SNF-related BAF chromatin remodeling complex subunit B1 (SMARCB1) expression [19]. It is sometimes difficult to distinguish UPS from HS with a spindled morphology. It should be noted, however, that UPS is negative for PU.1 in contrast to HS [20]. The corresponding histological and immunohistochemical characteristics are summarized in Table 1.

## 5. Pathogenesis

Only two cases of HS have cytogenetically been characterized in the literature [21,22]. One case showed a hyperdiploid karyotype (57–80 chromosomes), including 3 to 4 copies of various chromosomes [21]. The other case revealed 47XY, add(4)(p16), +8/48XY, add(4)(p16), +8, +8/48, XY, del(3)(q11), add(4)(p16), +8, +8/48, XY, t(3;5)(q25;q35), +8, +8 [22].

Mutations involving the RAS/mitogen-activated protein kinase (MAPK) signaling pathway have been detected in 57–90% of cases [23,24,25,26]. Mitogen-activated protein kinase kinase 1 (*MAP2K1*), B-Raf proto-oncogene, serine/threonine kinase (*BRAF*), and KRAS proto-oncogene, GTPase (*KRAS*) are the most frequently mutated genes in this pathway [23,24]. Moreover, another study found *BRAF* V600E mutations in 62.5% of cases [27]. In addition to these genes, recurrently mutated genes include neurofibromin 1 (*NF1*), NRAS proto-oncogene, GTPase (*NRAS*), protein tyrosine phosphatase non-receptor type 11 (*PTPN11*), and Cbl proto-oncogene (*CBL*) [23,24,25]. Moreover, high-level amplification of *PTPN11* has been found [25]. Interestingly, Egan et al. reported that cases with alterations in *NF1* and/or *PTPN11* had a predilection for GI tract involvement [25].

Mutations involving the phosphoinositide-3 kinase (PI3K)/AKT/mechanistic (formerly mammalian) target of the rapamycin (mTOR) signaling pathway have also been identified in 15–21% of cases [23,24,25]. Mutated genes in this pathway include phosphatase and tensin homolog (*PTEN*), *MTOR*, phosphoinositide-3-kinase regulatory subunit 1 (*PIK3R1*), phosphatidylinositol-4,5-bisphosphate 3-kinase catalytic subunit alpha (*PIK3CA*), and phosphatidylinositol-4,5-bisphosphate 3-kinase catalytic subunit delta (*PIK3CD*). Moreover, *PIK3CA* amplification has been observed in one case [24].

Tumor suppressor genes such as cyclin dependent kinase inhibitor 2A (*CDKN2A*) and tumor protein p53 (*TP53*) are most frequently altered genes [23,24]. *CDKN2A* mutations or deletions have been identified in 39–46% of cases [23,24]. Intriguingly, all cases with homozygous *CDKN2A* deletion revealed complete loss of p16 protein expression [23]. In addition, one study provided genetic evidence of the cooperative interactions of the *CDKN2A* and *PTEN* genes in the development of human and mouse HS [28]. *TP53* mutations have been detected in 24% of cases [24]. We speculate that mutations in *CDKN2A* and/or *TP53* may be associated with aggressive clinical features and poor survival of HS patients.

Alterations in colony stimulating factor 1 receptor (*CSF1R*) have been identified in 7–17% of cases [24,29], suggesting a potential therapeutic target. It has been known that activating *CSF1R* mutations are also found in other histiocytic neoplasms and increase MAPK pathway activity [29].

Alterations in lysine methyltransferase 2D (*KMT2D*) have been documented in some HSs, especially FL-associated secondary HS [25,26,30]. These findings indicate that the presence of recurrent *KMT2D* alterations implicates epigenetic regulation in the pathogenesis of HS.

In addition to somatic mutations, a variety of gene fusions have been discovered in HS, including tropomyosin 3 (*TPM3*)-neurotrophic receptor tyrosine kinase 1 (*NTRK1*), MYC proto-oncogene, bHLH transcription factor (*MYC*)-T cell receptor alpha locus (*TRA*), immunoglobulin heavy locus (*IGH*)-BCL2 apoptosis regulator (*BCL2*), *IGH*-BCL6 transcription repressor (*BCL6*), *BRAF*-myelin basic protein (*MBP*), *BRAF*-CAP-Gly domain containing linker protein 2 (*CLIP2*), *BRAF*-nuclear respiratory factor 1 (*NRF1*), tweety family member 3 (*TTYH3*)-*BRAF*, mitoguardin 2 (*MIGA2*)-*BRAF*, Rho GTPase activating protein 45 (*ARHGAP45*)-*BRAF*, and cyclin D1 (*CCND1*)-*IGH*, [23,24,25,30,31,32,33,34,35,36]. Of these, the most recurrent fusion is *IGH*-*BCL2*, which is a genetic hallmark for FL.

Transdifferentiation is commonly seen in HS. In 2008, Feldman et al. provided the first evidence of a clonal relationship between FL and HS with or without dendritic differentiation [37]. Subsequently, several authors have demonstrated cytogenetic/molecular evidence of a clonal relationship between primary B-cell lineage neoplasms and secondary or concurrent HS [32,38,39,40,41,42]. Although HS typically loses the immunomarkers of the related lymphomas, the underlying genetic alterations are preserved. Most recently, Seth et al. reported the first case of multiple myeloma transdifferentiating into HS, with cytogenetic and molecular confirmation of clonal relatedness [43]. These findings indicate that HS can arise as a result of transdifferentiation from hematolymphoid neoplasms, particularly low-grade B-cell lymphoma. In B cells, loss of paired box 5 (PAX5) expression is thought to be important in this process. Moreover, clonal *IGH* (±*IGK*) and T-cell receptor (TCR) gene rearrangements have been described in a subset of sporadic HS cases [36,44]. It is of particular interest that all 7 *IGH*/*IGK*-positive cases were negative for PAX5 and B-cell specific octamer binding protein-1 (BOB-1), whereas 4 (57%) of the 7 cases were positive for organic cation transporter 2 (OCT2) [36]. In daily clinical practice, it is important to look for concurrent or prior diagnosis of any type of lymphoma for HS cases.

Frequent genetic alterations observed in HS are summarized in Table 2.

## 6. Management

There is currently no standard protocol for the management of HS. In general, treatment of HS depends on the extent of disease and organs involved. Patients with disease involvement of critical organs such as CNS or liver are particularly difficult to manage. According to the National Cancer Database (NCDB) from 2004 to 2015, systemic chemotherapy alone was administered to 25% (82/330) of the patients, whereas 22% (74/330) had surgery alone and 4% (14/330) underwent radiotherapy (RT) alone [4]. Approximately 3% (10/330) of the entire cohort underwent hematopoietic stem cell transplantation (HSCT) [4].

### 6.1. Unifocal/Localized Disease

Surgical resection is the mainstay of treatment for patients with unifocal/localized HS, with a potentially curative purpose. The surgical procedure is wide resection with negative margins (R0, no microscopic residual tumor). However, achieving R0 resection can be more challenging for HS with vital organ involvement. Kommalapati et al. reported that surgical treatment with or without RT was associated with a prolonged OS than systemic therapy in patients with localized skin and connective tissue disease [4]. Currently, an adequate margin of resection for HS is not well established due to its infrequent occurrence and a limited number of reported cases.

Either alone or with systemic therapy, RT can play a critical role in the management of hematological malignancies [45]. In general, postoperative RT is considered for patients with positive or close surgical margins if re-resection is not possible. Although the optimal radiation dose in the adjuvant setting is unclear in HS, the use of adjuvant RT can reduce local recurrence rates [5,46,47]. Recently, Iyizoba-Ebozue et al. reported a case of localized HS involving the base of tongue that was successfully treated with radical RT (60 Gy in 30 fractions) as a single modality and achieved disease-free survival (DFS) beyond 5 years [48]. This case suggests that definitive RT may serve as an alternative treatment option for localized disease, particularly when the potential morbidity of surgery presents significant risks. In selected cases, palliative RT might be an option to give symptomatic relief [49]. To date, no data is available from randomized trials comparing surgery alone with the combined treatment of RT and surgery in HS. Further prospective randomized trials are required to better define optimal treatment approaches for unifocal/localized HS.

### 6.2. Multifocal/Disseminated Disease

The development of unresectable, disseminated or metastatic HS is associated with a very poor prognosis. Systemic therapy options for patients with multifocal/disseminated HS include chemotherapy, molecular targeted therapy, tyrosine kinase inhibitor (TKI), immunotherapy, and HSCT [4,6,50]. Currently, there is no regulatory-approved treatment for multifocal/disseminated HS, and multiple systemic agents are being used in the management of refractory/metastatic HS (Table 3).

Patients with advanced/metastatic disease usually receive multidrug chemotherapy used for diffuse large B-cell lymphoma (DLBL). Cyclophosphamide, hydroxydaunorubicin, oncovin, and prednisone (CHOP) with or without etoposide is the most commonly used regimen for multifocal/disseminated HS and can be used as a first-line treatment [6,51]. Several cases with complete or partial responses have been documented [51,52,53,54,55,56,57,58,59]. However, HS patients often relapse after CHOP and may require additional therapies such as second-line chemotherapy or HSCT. In addition to CHOP, ifosfamide, carboplatin, and etoposide (ICE) or adriamycin (doxorubicin), bleomycin, vinblastine, and dacarbazine (ABVD) has been used in clinical practice [51,60,61,62]. For instance, Hussein et al. described a 52-year-old man with HS involving multiple abdominal sites treated with surgical resection followed by 6 cycles of ICE chemotherapy, achieving significant clinical improvement and tumor regression [60]. On the other hand, Tomlin et al. reported a case of a 33-year-old man with disseminated HS who was treated with CHOP followed by salvage ICE, showing an unfavorable clinical course [62]. Subsequently, this patient received cladribine, high-dose cytarabine, G-CSF, and mitoxantrone (CLAG-M) and had a partial response (PR) with near resolution of disease. In 2017, Iwabuchi et al. reported a case of 8-year-old girl with primary HS involving the left femur who was treated with a combination of cladribine and high-dose cytosine arabinoside [63]. The patient remained in complete remission more than 7 years from diagnosis. As noted above, HS can arise subsequent to FL. Interestingly, Farris et al. reported a case of HS associated with FL who was treated with a combination of rituximab and bendamustine, achieving a complete radiological response [64]. Temozolomide (TMZ) is an oral alkylating agent used to treat high-grade glioma. A response to TMZ has been observed in one case of a 15-year-old girl with CNS HS [65]. The patient was treated with surgical resection with radiotherapy followed by 7 cycles of TMZ and remained recurrence-free without neurological deficit for 23 months. These results should be interpreted with caution because the number of cases is too small to make any definitive conclusion.

The discovery of *BRAF* and *MAP2K1* mutations has led to the development of target therapies that target the MAPK pathway. As mentioned above, *BRAF*, especially *BRAF* V600E, and *MAP2K1* mutations have been identified in a significant subset of HS cases. Vemurafenib, a BFAF inhibitor, has shown prolonged efficacy in patients with *BRAF* V600-mutant Erdheim-Chester disease (ECD) and Langerhans cell histiocytosis (LCH) [66]. A good response to vemurafenib has been observed in two HS cases displaying *BRAF* V600 mutations [67,68]. One case showed a dramatic clinical and radiological response to vemurafenib in a 40-year-old man with primary CNS HS harboring *BRAF* V600E mutations [67]. However, the response was not durable, and the patient died 6 months after initiation of vemurafenib treatment. The other case with *BRAF* V600E mutations was treated with vemurafenib after failure of etoposide, prednisone, vincristine, cyclophosphamide, and doxorubicin (EPOCH) chemotherapy, showing significant decrease in tumor size and metabolic activity [68]. In addition, dabrafenib (BFAF inhibitor) in combination with trametinib (MEK1/2 inhibitor) have also demonstrated sustained efficacy in patients with secondary HS harboring *BRAF* mutations [69,70,71]. Moreover, trametinib monotherapy has shown efficacy in patients with primary/secondary HS harboring *MAP2K1* or *KRAS*/*NRAS* mutations [72,73,74,75]. Voruz et al. reported a case of a 66-year-old man with multifocal HS harboring *PTPN11* mutations who was treated with trametinib after failure of intensive chemotherapy, achieving an excellent partial remission after 2 months of treatment [76]. The patient was subsequently treated with imatinib and then had a PR. Although the responses were generally durable, some patients experienced multiple adverse events and lost response after therapy interruption. In 2022, the United States Food and Drug Administration (FDA) approved cobimetinib (MEK1/2 inhibitor) for the treatment of adult patients with histiocytic neoplasms, including ECD, LCH, and Rosai-Dorfman disease (RDD) based on results from an open-label phase 2 trial (NCT02649972) [77]. Interestingly, Shanmugam et al. reported a dramatic clinical response and a partial radiological response to cobimetinib in a 39-year-old man with secondary *NF1*-mutated HS [23]. However, the optimal duration of targeted therapy for patients with HS and impact of treatment interruption remain unknown. A study of molecular targets for the treatment of histiocytosis (NCT04437381) is currently underway.

Sirolimus is an mTOR inhibitor and treatment with sirolimus and prednisone has been associated with a high rate of objective response (OR) in patients with multisystemic ECD [78]. As noticed above, 15–21% of HS patients demonstrate activation of the mTOR pathway. There are two case reports regarding the use of sirolimus in HS patients [70,79]. Chohan et al. reported a case of a 63-year-old woman with multifocal HS harboring *PTEN* mutations who was treated with sirolimus plus prednisone [79]. The patient had an objective clinical and radiological response for more than 12 months after sirolimus therapy. Venkataraman et al. reported that mTOR-direct therapy (one dose of temsirolimus and daily oral sirolimus) led to significant clinical and radiological improvement in a child with secondary HS harboring *MTOR* mutations [70]. Recently, Durham et al. reported clinical and metabolic complete remission to alpelisib (PI3K inhibitor) in a 46-year-old woman with *PIK3CA*-mutated LCH [80]. To date, however, there are no published reports concerning the efficacy of alpelisib in HS patients.

Alemtuzumab, a humanized monoclonal antibody targeting against CD52, is currently approved by the FDA for treatment of previously untreated patients with B-cell CLL. Single-agent alemtuzumab has been used in two CD-52 positive HS patients who were refractory to multiple lines of chemotherapy [81]. One patient had a complete response (CR) with no evidence of disease for more than 5 years, and the other had a major response with no evidence of disease for more than 4 years after alemtuzumab therapy. Recently, Valera et al. reported a case of 6-year-old boy with CD52-positive secondary HS who was treated with CHOP followed by alemtuzumab, demonstrating a short-term response [82].

Pexidartinib is an oral TKI with selective inhibition of CSF1R, which has been approved by the FDA for treatment of adult patients with symptomatic tenosynovial giant cell tumor (TSGCT) that is associated with severe morbidity or functional limitations and not responsive to improvement with surgery. As mentioned above, activating mutations in *CSF1R* have been identified in a subset of HSs. Although there are no published reports concerning the efficacy of pexidartinib in HS patients, it is of interest that at least two ECD patients with *CSF1R* mutations have been treated successfully with pexidartinib [83,84]. We speculate that pexidartinib may show clinical benefits in patients with refractory HS harboring activating *CSF1R* mutations.

Thalidomide, an oral agent with antiangiogenic and immunomodulatory properties, has shown efficacy in relapsed or refractory patients of multiple myeloma. There are a few case reports regarding thalidomide therapy of HS patients after failure of systemic therapy [85,86,87,88,89,90]. Thalidomide has been shown to be effective in salvage of refractory de novo HS when used alone or combined with chemotherapy [86,87,88]. In addition, thalidomide alone has contributed to long-term stabilization in a pediatric patient with secondary HS after allogeneic bone marrow transplantation for T-cell acute lymphoblastic leukemia (ALL) [85]. On the other hand, Ventura Aguiar et al. reported a case of a 56-year-old woman with disseminated HS who was treated with thalidomide and etoposide, showing no clinical response [89].

Allogeneic or autologous HSCT has been used as a treatment option in relapsed or refractory HS cases [59,61,62,86,87,91,92,93,94,95], but the data are limited to rare case reports and small case series with relatively short follow-up. Some patients have achieved complete remission with allogeneic HSCT [62,95] or autologous HSCT [87,92].

Immunotherapy has shown the potential to induce long-term remission in patients with refractory or relapsed hematological malignancies [96]. The major targets of FDA-approved immunotherapeutic antibodies are programmed cell death protein-1 (PD-1) and its ligand-programmed cell death, ligand-1 (PD-L1). Few studies have assessed the expression of PD-L1 in HS [68,97]. The expression rate of PD-L1 has been reported to be approximately 25–50% in HS. The prognostic value of PD-1/PD-L1 expression in HS remains unknown. Nivolumab and pembrolizumab are the two most representative PD-1 inhibitors that have already been approved by the FDA for treatment of patients with classical Hodgkin lymphoma. There are several case reports showing favorable outcomes with nivolumab [98,99,100,101] or pembrolizumab [94,101,102,103]. Bose et al. reported a case of a 17-year-old woman with metastatic HS who was treated with nivolumab after noting PD-L1 expression in 75% of the tumor cells, achieving a durable response [98]. Campedel et al. reported a case of a woman in her 60s with refractory HS displaying very high PD-L1 expression (95%) who was treated with nivolumab, showing a substantial response [99]. In 2023, Nguyen et al. reported a case of a 61-year-old man with metastatic HS who was treated with pembrolizumab after noting PD-L1 expression in 90% of the tumor cells and had a progression-free survival (PFS) exceeding 30 months [102]. Gao et al. reported a case of 66-year-old woman with refractory HS displaying very high PD-L1 expression (more than 90%) who was treated with pembrolizumab combined with gemcitabine, dexamethasone, and cisplatin (GDP) chemotherapy [103]. The patient remained in complete remission for over 4 years. Recently, Lin et al. reported a case of 45-year-old man with relapsed HS showing very high PD-L1 expression (90%) who was treated with nivolumab and pembrolizumab and had a PFS exceeding 3 years [101]. These results suggest that the responses to immune checkpoint inhibitor (ICI) therapy may be associated with the degree of PD-L1 expression. On the other hand, Furui et at. suggested that pembrolizumab may be effective for patients with PD-L1-positive HS, but the therapeutic effect may be limited and depend on the tumor microenvironment [104]. A phase 2 study of pembrolizumab in patients with histiocytic/dendritic cell neoplasms including HS and biologically selected subtypes of relapsed/refractory aggressive lymphomas (NCT03316573) is currently ongoing [105]. Most recently, Zhang et al. described a 58-year-old woman with HS concurrent with FL who archived long-term remission after a combination of Tislelizumab (PD-1 inhibitor) with Daratumumab (anti-CD38 monoclonal antibody), Pazopanib (TKI), and GDP chemotherapy [106]. Further studies are needed to verify the efficacy and safety of immunotherapy in patients with multifocal/disseminated HS.

**Table 3:** Current systemic therapies for multifocal/disseminated histiocytic sarcoma.

Treatment Modalities	Agents	Reference
Chemotherapy	CHOP (first-line treatment)	[51,52,53,54,55,56,57,58,59]
	ICE	[60,61,62]
	ABVD	[51]
	CLAG-M	[62]
	Temozolomide	[65]
Targeted Therapy	Vemurafenib (BRAF inhibitor)	[67,68]
	Dabrafenib (BRAF inhibitor)	[69,70,71]
	Trametinib (MEK1/2 inhibitor)	[69,70,71,72,73,74,75]
	Cobimetinib (MEK1/2 inhibitor)	[23]
	Sirolimus (mTOR inhibitor)	[70,79]
	Alemtuzumab (humanized anti-CD52 monoclonal antibody)	[81,82]
	Thalidomide (antiangiogenic and immunomodulatory properties)	[85,86,87,88,89,90]
	Nivolumab (PD-1 inhibitor)	[98,99,100,101]
	Pembrolizumab (PD-1 inhibitor)	[94,101,102,103]

CHOP: cyclophosphamide, hydroxydaunorubicin, oncovin, and prednisone; ICE: ifosfamide, carboplatin, and etoposide; ABVD: adriamycin (doxorubicin), bleomycin, vinblastine, and dacarbazine; CLAG-M: cladribine, high-dose cytarabine, G-CSF, and mitoxantrone; BRAF: B-Raf proto-oncogene, serine/threonine kinase; mTOR: mechanistic target of the rapamycin; PD-1: programmed cell death protein-1.

## 7. Conclusions and Future Directions

HS is an ultra-rare, highly aggressive hematopoietic neoplasm that can arise de novo or be secondary to other hematolymphoid neoplasms. It frequently occurs in extranodal sites such as skin and soft tissue and can be localized or disseminated. The diagnosis of HS often requires an extensive immunohistochemical panel to confirm the macrophage lineage and rule out other anaplastic neoplasms. Notably, HS expresses CD163, CD68, lysozyme, and PU.1, but S100 is negative, although focal weak reactivity can be seen in some cases. *MAP2K1*, *KRAS*, *BRAF* V600E or *CDKN2A* mutations are the most frequent genetic alterations in HS. Moreover, *BRAF* fusions have been identified in a subset of cases. Surgery is the preferred first-line intervention for unifocal/localized HS, although the use of RT in combination with surgery may be considered in appropriately selected patients. The management of multifocal/disseminated HS is extremely challenging. CHOP is most commonly used in the first-line treatment for multifocal/disseminated HS. Molecularly inspired therapeutics targeting the MAPK and PI3K/AKT/mTOR signaling pathways may provide clinical benefit in refractory/metastatic HS. ICIs may also be considered as salvage therapy in PD-L1-positive HS. Prospective and well-designed clinical trials are imperative to establish the best management of HS.

## Figures and Tables

**Table 1:** Histological and immunophenotypic characteristics of histiocytic sarcoma and its major differential diagnosis.

Neoplasm	Histological Features	IHC Markers
HS	Large round to oval cells with abundant eosinophilic cytoplasm, prominent inflammatory background	Histiocytic markers (CD163, CD68, lysozyme), PU.1, CD4, CD31, CD45, CD45RO, factor XIIIa
ALCL	“Hallmark” cells characterized by eccentric, horseshoe-or kidney-shaped nuclei, “doughnut” cells	CD30, T-cell markers (variable), negative for histiocytic markers
LCS	Langerhans cells with marked cytologic atypia and occasionally nuclear groove, coagulative necrosis, eosinophil-rich inflammatory background	CD1a, CD207, S100, CD68 (variable)
IDCS	Round, oval, or spindle cells with abundant pale-pink cytoplasm, characteristic paracortical involvement	S100 (diffuse, strong), CD68 (variable, weak)
FDCS	Spindle to ovoid cells with indistinct cell borders, abundant reactive small lymphocytes	CD21, CD23, CD35, CD68 (variable)
MS	Myeloid blasts (much smaller than HS cells with a higher N/C ratio)	CD13, CD33, CD68, MPO
Melanoma	Epithelioid and spindle cells with marked cytologic atypia, intracytoplasmic pigment	SOX10, HMB45, S100, typically negative for histiocytic markers
Carcinoma	Large epithelioid and round cells	Cytokeratins, EMA, typically negative for histiocytic markers
EPS	Large ovoid or polygonal epithelioid cells with abundant eosinophilic cytoplasm, plump spindle cells, prominent necrosis	Cytokeratins, EMA, SMARCB1 (expression loss), CD34 (variable)
UPS	Predominantly pleomorphic spindle cells	Negative for all distinct lineage markers

HS: histiocytic sarcoma; ALCL: anaplastic large cell lymphoma; LCS: Langerhans cell sarcoma; IDCS: interdigitating dendritic cell sarcoma; FDCS: follicular dendritic cell sarcoma; MS: myeloid sarcoma; EPS; epithelioid sarcoma; UPS: undifferentiated pleomorphic sarcoma; N/C: nucleus-to-cytoplasm; IHC: immunohistochemistry; MPO; myeloperoxidase; EMA: epithelial membrane antigen; SMARCB1: SWI/SNF-related BAF chromatin remodeling complex subunit B1.

**Table 2:** Frequent genetic alterations in histiocytic sarcoma.

Pathway/Function	Gene	Genetic Alterations	Frequency (%)	Reference
RAS/MAPK pathway	*MAP2K1*	Mutations	15–24	[23,24,25]
	*KRAS*	Mutations	12–50	[24,25,26]
	*BRAF* V600E	Mutations	62.5	[27]
	*NF1*	Mutations	7–29	[24,25]
	*NRAS*	Mutations	5–7	[24,25]
	*PTPN11*	Mutations	5–19	[24,25]
	*CBL*	Mutations	12	[24]
PI3K/AKT/MTOR pathway	*PTEN*	Mutations	7–12	[23]
	*MTOR*	Mutations	7	[23]
Cell cycle regulation	*CDKN2A*	Mutations/Deletions	39–46	[23,24]
DNA damage	*TP53*	Mutations	24	[24]
Receptor tyrosine kinase	*CSF1R*	Mutations	7–17	[24,29]
Epigenetic regulation	*KMT2D*	Mutations	18	[23]
Immunoglobulin	*IGH*	Rearrangements	39	[36]

MAPK: mitogen-activated protein kinase; PI3K: phosphoinositide-3 kinase; MTOR: mechanistic target of the rapamycin; MAP2K1: mitogen-activated protein kinase kinase 1; KRAS: KRAS proto-oncogene, GTPase; BRAF: B-Raf proto-oncogene, serine/threonine kinase; NF1: neurofibromin 1; NRAS: NRAS proto-oncogene, GTPase; PTPN11; protein tyrosine phosphatase non-receptor type 11; CBL: Cbl proto-oncogene; PTEN: phosphatase and tensin homolog; CDKN2A: cyclin dependent kinase inhibitor 2A; TP53: tumor protein p53; CSF1R: colony stimulating factor 1 receptor; KMT2D: lysine methyltransferase 2D; IGH: immunoglobulin heavy locus.

## Data Availability

No new data were created or analyzed in this study.
